# Comparison of the Adult Strabismus Quality of Life Questionnaire (AS-20) with the Amblyopia and Strabismus Questionnaire (ASQE) among adults with strabismus who seek medical care in China

**DOI:** 10.1186/1471-2415-14-139

**Published:** 2014-11-22

**Authors:** Zonghua Wang, Hui Ren, Rosemary Frey, Yang Liu, Deborah Raphael, Wei Bian, Xianyuan Wang

**Affiliations:** School of Nursing, Third Military Medical University, Shapingba District, Chongqing, 400038 China; School of Nursing, University of Auckland, Auckland, New Zealand; Southwest Eye Hospital, Third Military Medical University, Shapingba District, Chongqing, 400038 China

**Keywords:** Quality of life, Adult strabismus, Questionnaire, Chinese

## Abstract

**Background:**

The impact of strabismus on visual function, self-image, self-esteem, and social interactions might decrease health-related quality of life (HRQoL). This study aimed to evaluate the psychometric properties and clinical applications of two strabismus-specific HRQoL questionnaires in the cultural context of China.

**Methods:**

The Chinese versions of the Adult Strabismus Quality of Life Questionnaire (AS-20) and the Amblyopia and Strabismus Questionnaire (ASQE) were self-administered to 304 adults with strabismus. The Cronbach’s α coefficient was calculated to assess the internal consistency reliability. The criterion-related validity was identified by exploring Spearman’s correlation with the most widely used vision-specific quality of life questionnaire NEI-VFQ-25. One-way ANOVA was employed to examine the differences in the quality of life of strabismus patients with visually normal adults and with other eye diseases patients.

**Results:**

Significantly positive correlations with NEI-VFQ-25 were shown in both scales (r = 0.21 - 0.44, p <0.05, p <0.01). Both scales could distinguish individuals with strabismus from visually normal adults (p <0.001) and adults with other eye diseases (p <0.001). The overall Cronbach’s α value were 0.91 for the AS-20 and 0.89 for the ASQE; and for the subscales, the α value ranged from 0.68 to 0.90.

**Conclusion:**

This was the first cross-sectional study to compare the psychometric properties of two strabismus-targeted questionnaires, AS-20 and ASQE in the context of Chinese culture. Both AS-20 and ASQE showed satisfactory and comparable properties for measuring HRQoL in strabismus patients.

## Background

The concept of health-related quality of life (HRQoL) has gained increasing popularity in clinical settings, either as a reference for making healthcare interventions or an index for evaluating medical treatment outcomes [1–3]. HRQoL is one aspect of quality of life specifically associated with the field of human health. WHO defines quality of life (QoL) as an “individuals’ perception of their position in life in the context of the culture and value systems in which they live and in relation to their goals, expectations, standards and concerns” [4]. Six fundamental dimensions are suggested to be included in HRQoL: physical, social, and psychological functioning, role activities, overall life satisfaction, and perceptions of health status [5, 6]. HRQoL differs across groups of patients as defined by disease, levels of severity, demographic features, socioeconomic status and cultural background. Therefore increasing awareness has been placed on developing disease-specific HRQoL instruments and applying to research studies [7]. Disease-specific questionnaires include items that are designed to be relevant to a particular group of patients, and are therefore more sensitive to small but clinically significant changes in disease and related quality of life either in screening or after treatment compared to a generic instrument [8–10].

The importance of HRQoL among adults with strabismus has been underestimated in China. Strabismus which may accompany amblyopia might decrease HRQoL by resulting in visual dysfunction, self-image disorders, low self-esteem, and social and emotional barriers [11, 12]. Individuals with strabismus fail to achieve proper binocular vision because they are unable to simultaneously direct each eye to the same point in space. In addition, the appearance of misaligned eyes might result in social prejudice by associating strabismus with personality defects and below average intelligence [13, 14]. Prejudice relating to strabismus can extend beyond social relationships. Adults with strabismus are likely to develop mannerisms to camouflage their dysfunction and avoid eye contact during social interactions [14, 15]. They also perceive that strabismus has a negative impact on secure employment and opportunities for career advancement [13].

Two strabismus-specific questionnaires for adult patients have been developed and applied in clinical settings and research studies [16]: the Adult Strabismus Quality of Life Questionnaire (AS-20) and the Amblyopia and Strabismus Questionnaire (A&SQ). The A&SQ was originally designed in Dutch [17], and subsequently translated into English (the English-language version of the Amblyopia and Strabismus Questionnaire, ASQE) [18, 19]. Several commonalities are shared by the AS-20 and ASQE. Both instruments utilized a qualitative-deductive method to develop questionnaire items by exploring patients’ own complaints and concerns. Answers for each item are recorded in a Likert-type rating scale: never (score 100), rarely (75), sometimes (50), often (25), and always (0). The composite score is derived from the mean of all the questions answered. Mean scores on both questionnaires range from 0 to 100 with higher scores indicating better quality of life. Finally, both measures are self-administered and consist of a small number of items (below 30 total items), which facilitate administration in time-sensitive clinical settings. However, the two questionnaires differ from each other regarding the development of items, subjects, and content (Table 1).

**Table 1:** **Summary of differences between AS-20 and ASQE**

Scale	Subjects	Item and subscale development	Domains or subscales (items)
AS-20	Adults with strabismus	1. A 181-item questionnaire was first generated on the basis of the patients’ interviews;	Psychosocial (10)
		2. 49 items were left for factor analysis after testing among 29 adult strabismus patients, and 2 factors were isolated: 1^st^ psychosocial functioning and self-awareness and 2^nd^ physical and emotional functions;	Function (10)
		3. 10 items with the highest loading were selected for each factor and a final 20-item questionnaire was established referring to Cronbach’s αcoefficient.	
ASQE	Adults with amblyopia and strabismus	1. An inventory of all problems that amblyopia and strabismus patients experienced were collected from outpatients and reduced to 5 domains;	Fear of losing better eye (2) Distance estimation (10)
		2. Then a final questionnaire was established after 26 questions based on these domains were formulated;	Visual disorientation (3) Diplopia (4)
		3. These questions were chosen from a pool of situations based on three criteria: 1) the best reflection of restrictions in daily functions; 2) description of only one situation in one question; 3) all interviewees could answer.	Problems with social contact and cosmetic problems (4)

Extracted and summarized from the following articles:

Graaf et al. [17].

Hatt, et al. [21].

According to the above WHO’s conceptualization of QoL, the cultural context in which people live should be considered when measuring HRQoL, since different cultural groups may vary in both understanding and expression of health and related QoL [20]. This consideration becomes extremely important when conducting multi-national or cross-cultural studies. However, as far as we know, there has been no culturally appropriate questionnaire for measuring HRQoL among strabismus patients in China. Since AS-20 and ASQE have shown high reliability and validity and have been applied widely in clinical practices [1, 2, 19] and research studies [21–23] across English-speaking countries, our study team sought to translate and validate both instruments within the context of Chinese culture rather than create a new questionnaire. The aims of this study were to compare the psychometric properties and clinical applications of the Chinese versions of the AS-20 and ASQE.

## Methods

### Ethical consideration

Ethical approval was obtained from the human ethics committee of the first affiliated hospital of Third Military Medical University. A participant information sheet and verbal explanation were given. This is an anonymous questionnaire, so no personally identifiable information was recorded. The consent to participate in this survey was assumed upon the completion of this questionnaire (i.e., completing the questionnaires implies giving consent to participate). All study procedures were conducted in accordance with the Declaration of Helsinki.

### Participants

A consecutive sample of 331 adult patients with strabismus was invited at Southwest eye hospital (Chongqing, China). They were attending the hospital in connection with their strabismus, possibly with a view to having strabismus surgery. After examination by ophthalmologist, patients who met surgical indications were added to a waiting list for a strabismus surgery. In this study, inclusion criteria for strabismus patients were: 1) aged 18 years and over; 2) no history of any eye-related surgery before participation nor any diagnosed emotional disorders; 3) no other facial or ocular abnormalities or acute eye diseases; 4) visual acuity ≥20/50 in the better-seeing eye; and 5) the angle of deviation by prism at distance was no less than 15PD. Thirteen patients refused to participate, yielding a response rate of 96.1%.

A control group of 100 adults without any visual defect and another control group of 100 patients with other eye diseases were recruited (both groups are orthotropic adults). They were all from the same eye outpatient clinic as the adult strabismus patients. Visually normal adults were companions or family members of the patients in the eye clinic. In visually normal adults, stereo acuity was examined by Titmus test (median, 40 seconds of arc); and visual acuity in the better-seeing eye was at least 20/25 (median, 20/20 in each eye). In the ‘other eye diseases’ patients, diagnoses were: retinal detachment (n = 23), vitreous haemorrhage (n = 18), cataract (n = 30), glaucoma (n = 19), and ocular trauma (n = 10); and visual acuity ranged from 20/20 to 20/40 (median, 20/30) for the better eye and from 20/20 to 20/80 (median, 20/40) for the worse eye.

### Questionnaires

The Chinese versions of the AS-20 and the ASQE have been developed following standard processes of translation and adaptation [24]. Specifically, both instruments were translated from English to simplified Chinese by two medical postgraduate students whose first language was Mandarin, and who also spoke English fluently. The instruments were then back translated to English by another two healthcare-related bilingual speakers. Discrepancies were discussed and resolved by an expert panel consisting of one ophthalmologic doctor, two ophthalmologic nurses, one charge nurse and one psychology professor. The validity and reliability of the Chinese version of the AS-20 [25] and the ASQE were satisfactory.

The 25-Item National Eye Institute Visual Function Questionnaire (NEI-VFQ-25), one of the most widely used vision-specific instruments for assessing both self-reported visual functions and vision-related quality of life among people with eye diseases, was translated and culturally adapted from English to Chinese by Wang and associates [26]. It contained 25 items in 12 subscales: general health, general vision, ocular pain, near activities, distance activities, vision-specific domains (i.e., social function, mental health, role difficulties, and dependency), driving, color vision, and peripheral vision. Each item was scored on a 5 level Likert-scale from 0 to 100. The total score was calculated as the average of all items responded except for the ‘general-health’ question, which was treated as a stand-alone item. The reliability of the Chinese version of NEI-VFQ-25 was satisfactory, with Cronbach’s α value ranging from 0.73 to 0.87 [26].

### Data collection

All data were collected among strabismus patients prior to any strabismus-related surgery. To guarantee the quality of response, verbal and written instructions were given by researchers before the participants were left alone in a reception room to complete the questionnaires. Researchers emphasised that participation was entirely voluntary and the choice to participate or not had no impact on their surgery or treatment.

For the purpose of standardisation, the questionnaires were bound in a fixed order: the first page was the AS-20, and then followed by the ASQE in the second and third pages. A subgroup of 93 strabismus patients was randomly selected to fill the NEI-VFQ-25 after completing the AS-20 and ASQE. Demographic information, diagnosis, presence of amblyopia and diplopia, and deviation size were also collected. Strabismus patients with amblyopia was defined as: the best corrected visual acuity ≤20/25, or interocular difference in visual acuity of ≥2 lines [18].

### Statistical analysis

The software IBM SPSS (Version 20.0) and GraphPad Prism for Windows were used for data analysis. A p value of 0.05 was adopted as the level of statistical significance. Since the HRQoL data were not normally distributed, Spearman’s rank correlation coefficient was employed for assessing the associations between the scores of AS-20 and ASQE with those of NEI-VFQ-25. The correlation coefficient is defined as follows: *r* <0.30 stands for a weak correlation and little clinical applicability, even when statistically significant; *r* ranging between 0.30 - 0.50, moderate and *r* >0.50, strong correlation [27, 28]. We compared the mean score in adult strabismus patients, with that in visually normal adults and in patients with other eye diseases using one-way ANOVA and Tukey's Multiple Comparison Test.

Reliability refers to the ability of an instrument to measure consistently. Internal consistency is one of the most commonly used indices of reliability that describes the extent to which a set of items in a test measure the same concept or construct the correlations between different items on the same test (inter-correlations of the items within the test) [29]. Internal consistency is usually assessed by Cronbach's α value, a statistic calculated from the pairwise correlations between items. It is expressed as a number between 0 and 1. George and Mallery [30] defined a Cronbach’s α value ≥0.90 as excellent internal consistency; value ≥0.80 as good and ≥0.70 as acceptable, while a value <0.70 indicates questionable internal consistency, poor (<0.60) and unacceptable (<0.50). When an α value <0.70 was obtained, one might doubt whether all items measure the same construct. If an α value increases when one item is removed, then this item might be considered to be amended or deleted; otherwise, if removing one item results in no increase in α value, implying that this item correlates well with other items to test the same construct [21]. The subscales with more than 20% of total strabismus patients responding a minimum/maximum score (indicating floor or ceiling effects) were also examined [26].

## Results

### Demographic characteristics

A total of 318 questionnaires were distributed, of which four were not returned and ten were not fully or correctly completed. Thus 304 valid questionnaires were available for statistical analysis (mean age, 26.8 ± 9.0 years; range, 18–67). Demographic information and clinical features of the strabismus patients were shown in Table 2. One hundred and sixty-two (53.3%) were male. Only 73 (24.0%) of the patients hold a university certificate and above, while 117 (38.5%) reported an education level below secondary school. Forty (13.2%) adult patients with strabismus never received any family support, and 189 (62.2%) did not receive any form of health insurance support. Family support in this study is inclusive of both financial and emotional support. No significant influences of demographic factors on HRQoL were reported with the exception of family support. Strabismus patients who never received any family support reported significantly worse HRQoL scores than those who ‘sometimes’ and ‘always’ received family support in the AS-20 overall scale (p < .001) as well as the two subscales of psychosocial (p = .014) and function (p < .001); however there was no significant difference in the ASQE (p values ranging from 0.083 to 0.355 for the overall scale and subscales).

**Table 2:** **Overview of demographic features and clinical characteristics**

	Strabismus adults n (%)
**Gender**	
Male	162 (53.29)
Female	142 (46.71)
**Family support**	
Never	40 (13.16)
Sometimes	104 (34.21)
Always	160 (52.63)
**Education level**	
Middle school and lower	117 (38.49)
High school	114 (37.50)
University degree and higher	73 (24.01)
**Socioeconomic status**	
Urban	122 (40.13)
Rural	182 (59.87)
**Health insurance support**	
Yes	115 (37.83)
No	189 (62.17)
**With double vision/Diplopia**	
Yes	100 (32.89)
No	204 (67.11)
**Types of strabismus**	
Esotropia	137 (45.07)
Exotropia	167 (54.93)
**Deviation size**	
≤ 25 PD	66 (21.71)
> 25 PD	238 (78.29)
**With Amblyopia**	
Yes	131 (43.09)
No	173 (56.91)

### Clinical features and HRQoL

**Figure 1:**
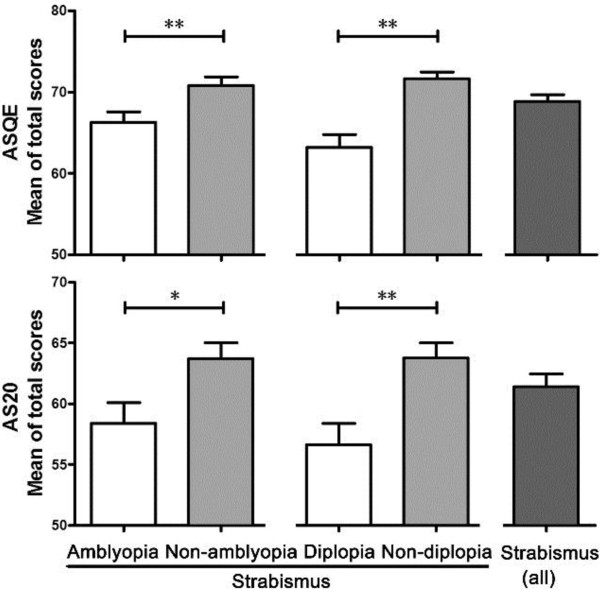
**The effects of strabismus on HRQoL in patients with and without amblyopia/diplopia.** This figure was derived from Independent t- test (*p <.05, 2-tailed, **p <.01, 2-tailed), aiming to interpret the effects of strabismus on quality of life in patients with and without amblyopia/diplopia.

One third (32.9%) of strabismus patients self-reported a symptom of double vision, and 137 (45.1%) were diagnosed as esotropia. No patients with vertical strabismus were reported in this study cohort. The group with exotropia recorded better HRQoL scores than patients with esotropia in the overall AS-20 (p = 0.003), the psychosocial subscale (p = 0.002), the function subscale (p = 0.035) and in the ASQE subscale of ‘social contact and appearance’ (p = 0.015). Except for the subscales of ‘psychosocial’ (p = 0.390) and ‘fear of losing the better eye’ (p = 0.146), strabismus patients with diplopia reported significantly lower HRQoL scores in the overall AS-20 (p = 0.001) and ASQE (p <0.001) (Figure 1) and all other subscales (all p values ≤0.001 excluding the subscale of ‘social contact and appearance’ with p = 0.021). Compared to strabismus patients without amblyopia, significantly lower HRQoL scores were recorded among those with amblyopia in total AS-20 (p = 0.012) (Figure 1) and in the ‘psychosocial’ subscale (p = 0.011). The same pattern was found in the overall ASQE (p = 0.005) (Figure 1), and the subscales of ‘fear of losing the better eye’ (p = 0.026), ‘diplopia’ (p = 0.009) and ‘social contact and appearance’ (p = 0.012). No significant differences of deviation size on HRQoL scores were identified in this study.

### Comparison with control groups

**Figure 2:**
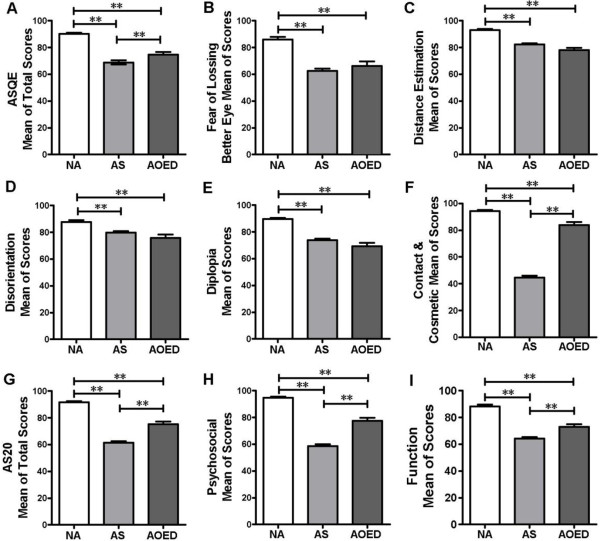
**Comparison of mean of strabismic adults versus visually normal adults and patients with other eye diseases. A**. the overall ASQE. **B**. fear of losing better eye. **C**. distance estimation. **D**. disorientation. **E**. diplopia. **F**. contact & cosmetic **G**. the overall AS-20. **H**. psychosocial. **I**. function. This histogram was derived from one-way ANOVA and Tukey's Multiple Comparison Test (**P <.001, 2-tailed), aiming to interpret the comparisons of average health-related quality of life scores among visually normal adults, adult patients with strabismus and adults with other eye diseases. NA, normal adults; AS, adults with strabismus; AOED, adults with other eye diseases.

No statistically significant differences were found between the study groups (strabismus patients, visually normal adults, and patients with other eye diseases) in terms of age, gender, education, and socioeconomic status. The overall mean scores for the AS-20 and both subscales were significantly lower in strabismus patients compared to those in the visually normal group and the other eye diseases group (p <0.001) (Figure 2G-I). When using ASQE, adults with strabismus showed significantly lower quality of life scores in comparison to individuals with normal vision in the overall scale and all subscales (p <0.001) (Figure 2A-F), while significant differences were only reported in the composite score and the subscale of “social contact and appearance” between the patients with strabismus and adults with other eye diseases (p <0.001) (Figure 2A and F).

### Correlation with NEI-VFQ-25 scores

Weak to moderate but statistically significant correlations of the AS-20 and the ASQE with NEI-VFQ-25 have been found, particularly for the mean scores of the total scale (r = 0.21 - 0.34, p <0.05 or p <0.01), ‘general health’ (r = 0.20 - 0.31, p <0.05 or p <0.01) and ‘vision-specific domain’ (r = 0.25 - 0.44, p <0.05 or p <0.01) (Table 3). Overall, the ASQE and each of its subscales recorded a stronger correlation with the NEI-VFQ-25 in comparison to the AS-20.

**Table 3:** **Correlation of AS-20 and ASQE with NEI-VFQ-25 on total score and subscales**

NEI-VFQ-25	AS-20			ASQE					
	Total	Psychosocial	Function	Total	FL	DE	VD	DV	SA
Total Score	**0.209** ^*^	0.137	**0.218** ^*^	**0.391** ^**^	**0.241** ^*^	**0.280** ^**^	**0.294** ^**^	**0.393** ^**^	0.007
General Health	**0.255** ^*^	0.155	**0.305** ^**^	**0.323** ^**^	**0.204** ^*^	**0.256** ^*^	**0.204** ^*^	**0.308** ^**^	0.096
General Vision	0.104	0.018	0.158	0.202	**0.216** ^*^	0.105	0.096	0.135	-0.042
Ocular Pain	0.192	0.125	**0.206** ^*^	0.200	0.119	0.131	0.164	0.184	-0.038
Near Activities	-0.054	-0.058	-0.068	0.062	0.062	0.067	0.062	0.165	**-0.223** ^*^
Distance Activities	0.108	0.037	0.134	**0.348** ^**^	**0.249** ^*^	**0.354** ^**^	**0.214** ^*^	**0.398** ^**^	-0.044
Vision-specific	**0.343** ^**^	**0.310** ^**^	**0.272** ^**^	**0.425** ^**^	0.165	**0.345** ^**^	**0.254** ^*^	**0.439** ^**^	**0.218** ^*^
social functioning	0.203	**0.218** ^*^	0.082	**0.252** ^*^	-0.018	**0.279** ^**^	**0.221** ^*^	**0.315** ^**^	0.147
mental health	**0.361** ^**^	**0.318** ^**^	**0.311** ^**^	**0.476** ^**^	**0.212** ^*^	**0.381** ^**^	**0.224** ^*^	**0.437** ^**^	**0.267** ^**^
role difficulties	0.174	0.144	0.146	**0.278** ^**^	0.126	**0.222** ^*^	**0.219** ^*^	**0.325** ^**^	0.049
dependency	**0.357** ^**^	**0.297** ^**^	**0.323** ^**^	**0.414** ^**^	0.156	**0.348** ^*^	0.198	**0.434** ^**^	**0.237** ^*^
Driving	0.082	0.043	0.073	0.191	0.228	0.137	-0.018	**0.247** ^*^	0.066
Color Vision	0.175	0.149	0.155	0.193	0.075	0.108	**0.246** ^*^	0.197	0.043
Peripheral Vision	-0.001	-0.039	0.008	**0.219** ^*^	0.092	0.194	**0.274** ^**^	**0.251** ^*^	-0.017

FL, fear of losing the better eye; DE, distance estimation; VD, visual disorientation; DV, double vision; SA, social contact and appearance.

Derived from the Spearman rank correlation coefficient *r*.

Significant correlations are listed in bold (^*^ *p* <0.05, 2-tailed; ^**^ *p* <0.01, 2-tailed).

### Internal consistency reliability

Internal consistency reliability for both the AS-20 and the ASQE were satisfactory (Cronbach’s α ≥0.70) except for the subscale of the ASQE ‘double vision’ (α = 0.68) (Table 4). The overall Cronbach’s α value was 0.91 for the AS-20 and 0.89 for the ASQE. Specifically, the Cronbach’s α values were 0.90 and 0.85 for the ‘psychosocial’ (10 items) and ‘function’ (10 items) subscale, respectively; and for the ASQE subscales, the α values were 0.89 for ‘fear of losing the better eye’ (2 items), 0.86 for the ‘distance estimation’ (10 items), 0.88 for the ‘visual disorientation’ (3 items), and 0.76 for the ‘social contact and appearance’ (4 items).

**Table 4:** **Internal consistency reliability of the AS-20 and the ASQE**

Subscale	Number of items	Internal consistency reliability*
AS-20		
Total	20	0.91
Function	10	0.90
Psychosocial	10	0.85
ASQE		
Total	26	0.89
Fear of losing the better eye	2	0.89
Distance estimation	10	0.86
Visual disorientation	3	0.88
Double vision	4	0.68
Social contact and appearance	4	0.76

*Cronbach’s α coefficient.

### Floor/ceiling effects

A floor effect (>20% of scores at the minimum score) was only detected in the ‘social contact and appearance’ subscale, with 21.5% of strabismic patients reporting the lowest score. In contrast, all subscales except the ‘social contact and appearance’ showed a ceiling effect (>20% at the maximum score), with a percentage of patients with the highest scores between 24.1% for the ‘psychosocial’ subscale and 62.7% for the “distance estimation” subscale (Table 5).

**Table 5:** **Floor/ceiling effects of the subscale scores of the AS-20 and ASQE**

Subscale	Floor (%)	Ceiling (%)
AS-20		
Psychosocial	10.16	*24.11*
Functional	8.78	*33.32*
ASQE		
Fear of losing better eye	8.39	*32.70*
Distance estimation	3.26	*62.70*
Visual disorientation	1.75	*49.23*
Double vision	5.43	*45.48*
Social contact & appearance	*21.46*	15.21

All values in italic type indicate floor effect or ceiling effect.

(>20% of scores at the minimum/maximum score).

## Discussion and conclusions

Consistent with previous studies, our results indicated that patients with diplopia would more often report function-related concerns over psychosocial effects. Felius and colleagues for example, found a negative association between diplopia and functional defects: the subscale scores of ‘distance estimation’ (r = -0.37, p <0.001), ‘visual disorientation’ (r = -0.17, p = 0.04) and ‘double vision’ (r = -0.51, p <0.001) were all correlated with the status of diplopia while the subscales ‘fear of losing the better eye’ and ‘social contact and appearance’ were not [18]. In contrast, Hatt et al. found high levels of psychosocial concern in some patients with diplopia [21]. For example, 76 (76%) and 89 (89%) of 100 patients with diplopia rated either ‘sometimes’, ‘often’ or ‘always’ on the question 17 (I feel stressed because of my eyes) and 18 (I worry about my eyes), respectively. Adult patients with diplopia often complain about difficulties to concentrate and to orientate [21]. They have to close one eye to see things better, but this behaviour can result in feelings of eye strain. This may explain why diplopic patients also report psychosocial concerns since double vision may make them feeling tired, stressed and worried. In regards to stabisimus patient, ophthalmologist and nurses should observe not only the evidence of difficulties with daily visual functions but also related psychosocial concerns.

Another factor that may influence the quality of life among strabismus patients is the presence of amblyopia. Our results showed that strabismus patients with amblyopia scored lower than those without amblyopia. Amblyopia was expected to be closely associated with functional difficulties, such as weak vision and loss of stereopsis [31]. But interestingly, in this study, the difference in psychosocial concerns (fear of losing the better eye, appearance and difficulties with social contact) were greater between strabismic patients with and without amblyopia. It could be speculated that adult strabismus patients already have adjusted to living with functional difficulties due to amblyopia (for example, compensate the better eye for seeing things) and accepted the low likeliness for vision improvement. Therefore, they abandoned the expectations for improved eye sight and shift attention to their appearance and social relationship which might be greatly improved after a surgery.

We found that both the AS-20 and the ASQE showed significant differences between visually normal adults and strabismus patients in terms of total scores as well as all subscale scores; however, significant differences were only found between adults with other eye diseases and with strabismus for the ASQE overall scale and the subscale “social contact and appearance”, and for the AS-20 overall scale and both subscales. Besides, it showed that the magnitude of the differences in mean scores between adults with normal vision and with strabismus was greater than those between individuals with other eye diseases and with strabismus (Figure 2). These results may suggest that the AS-20 is capable of detecting smaller differences compared with the ASQE. It should be noted that although the ASQE distributes the HRQoL into more dimensions while the AS-20 only summarises two subscales of “psychological” and “function”, a closer examination of the items within the AS-20 indicates similar content to those in the ASQE. For example, in the AS-20 item 9 (people react differently to me because of my eyes) could be categorised under ‘social contact’ and item 14 (I have problems with depth perception) would fit under ‘distance estimation’.

In the previous studies, the Cronbach’s α values of the AS-20 and its subscales were reported ranging from 0.94 to 0.95 by Hatt and colleagues [21]; and that of the original A&SQ and all subscales were from 0.76 to 0.93 [17], while the English version ASQE presented a range between 0.80 and 0.92 [18]. In this study, the internal consistency reliability of both instruments was satisfactory except for the subscale “double vision”. Similarly, Van de Graaf et al. [17] found that the reliability of the domain “double vision” increased after deleting the last three questions from the final analysis. When we looked back the content of the ASQE, we could see that except for the item 18 which directly related to “double vision”, the rest of the items (questions 19 to 21) were describing some other functional status beyond double vision. Therefore one possible explanation for the relatively low internal reliability of the domain “double vision” is that the proposed function descriptions found in these three items were not exclusively features of diplopia. For example, patients with low visual acuity may also do things more slowly or be more careful not to miss what they reach for when they are tired (item 20). The status described in the item 21 “I have to squint or shut one eye in bright sunlight” could also exist in visually normal adults when the sunlight is too bright. However, Felius et al. found an acceptable Cronbach’s α value of 0.82 [18] for this domain in ASQE among 150 adults from an English-speaking country. Since the original A&SQ was established and validated in a Dutch-speaking country, the factor of cultural difference need to be considered in.

A floor effect was detected in the “social contact and appearance” subscale. This meant that more than 20% of the strabismus patients had responded “always” to the items of this subscale, representing the lowest level of quality of life. This might be explained by the fact that the sample in this study was a selected population who have sought correction surgery as most of them were unhappy or dissatisfaction about their asymmetric appearance. A ceiling effect suggested more than 20% of the strabismus patients had reported “never” to items, representing the highest level of quality of life. Considering cultural difference, one possible explanation may be that the patients’ perception of, and response to, illness were closely associated with sociocultural factors [26]. Chinese people tend to under-report their health conditions [32]. Since the AS-20 and ASQE were both developed in the context of western culture, some items in the subscales may not apply to Chinese strabismus patients. As indicated by responses, they might have ‘never’ experienced some situations described in the items. Future research should include interviews or group discussions to explore what Chinese strabismus patients say about their strabismus-related quality of life.

In recent years an increasing number of health science researchers have advocated the Rasch model for analyzing the psychometric properties of a measurement [33]. Unlike classical test theory (CTT) which explores the difference between raw scores, Rasch analysis is one of the contemporary psychometric methods exploring the probability of an individual’s response to an item. This probability is defined by the latent trait (e.g., ability) of individual subjects and the difficulty of each item on the instrument being used [34]. While CTT focuses on issues of internal consistency, reliability and validity of a measurement; the Rasch model pays more attention to the essential features of a questionnaire, including unidimensionality, hierarchical order, and equal interval scaling. These features are essential for the measurement in order to make meaningful comparisons (e.g., to compare between patients; and to make comparisons over time) [35]. The previous studies on Rasch analysis of the AS-20 and ASQE suggested that some items should be removed [34, 36]. Nevertheless, in this study, we utilised the full form of both questionnaires to take account of cultural differences. In other words, the items that the Rasch analysis suggested for removal might apply to adult strabismus patients in China. It was therefore worthwhile to conduct a Rasch analysis of the AS-20 and ASQE among Chinese strabismus patients. Since Rasch analysis includes many parameters and is quite different from the CTT, a separate article is required for reporting these results.

As is the case with any research, some limitations must be acknowledged. Since our study patients were all recruited from an eye hospital, they potentially were more likely to be concerned about their eyes and have low HRQoL than those who did not attend. This may reduce the generalizability of the results to all members of the strabismus population. Moreover, it should be noted that the patients in our cohort were very young (mean age, 26.8 ± 9.0 years) compared to the previous studies (median age, 44 to 53 years) [21, 22] which could introduce a bias in the results. One possible explanation for this age difference relates to the strong emphasis Chinese people place on maintaining high self-esteem [37, 38]. Fears about losing esteem and feeling inferior to others because of strabismus and the associated poor cosmetic appearance may be a driving factor. Approaching doctors for strabismus treatment at an early age could help regain self-confidence and promote self-development in social networks and career life. This assumption can be supported by our study results revealing that significantly lower mean scores were obtained among strabismus patients in the subscale of “social contact and appearance” compared to visually normal adults and individuals with other eye diseases (Figure 2F). Further studies are needed to explore qualitative information and to examine whether self-confidence and HRQoL is improved after strabismus treatment. In spite of the above limitations, this study is the first cross-sectional study to compare psychometric parameters between strabismus-targeted questionnaires AS-20 and ASQE in the context of Chinese culture. The study results are of relevance to healthcare professionals who work with those strabismus patients in eye hospitals. The study also provides evidence to support the application of HRQoL as an index for assessing the needs of strabismus patients and evaluating treatment outcomes in clinical practices.

In conclusion, both the AS-20 and ASQE presented comparable properties for measuring quality of life in strabismus patients who seek medical care. Both scales were vision-targeted and showed satisfactory and acceptable reliability. The overall Cronbach’s α values were 0.91 for the AS-20 and 0.89 for the ASQE; and ranged from 0.68 to 0.90 for the subscales. Both of the scales distinguished individuals with strabismus from visually normal adults and patients with other eye diseases, but the AS-20 revealed better sensitivity. In addition, future work should look at which of the two instruments is more patient-friendly and appropriate to administer.
